# Evodiamine suppresses non-small cell lung cancer by elevating CD8^+^ T cells and downregulating the MUC1-C/PD-L1 axis

**DOI:** 10.1186/s13046-020-01741-5

**Published:** 2020-11-19

**Authors:** Ze-Bo Jiang, Ju-Min Huang, Ya-Jia Xie, Yi- Zhong Zhang, Chan Chang, Huan-Ling Lai, Wenjun Wang, Xiao-Jun Yao, Xing-Xing Fan, Qi-Biao Wu, Chun Xie, Mei-Fang Wang, Elaine Lai-Han Leung

**Affiliations:** 1 State Key Laboratory of Quality Research in Chinese Medicine, Macau University of Science and Technology, Avenida Wai Long, Macao, Taipa Macau (SAR) China; 2 Department of Respiratory and Critical Care Medicine, Taihe Hospital, Hubei University of Medicine, Shiyan, 442000 China; 3 Hubei Key Laboratory of Embryonic Stem Cell Research, Shiyan, China

**Keywords:** Evodiamine, PD-L1, MUC1-C, NSCLC, Immune microenvironment

## Abstract

**Background:**

Accumulating evidence showed that regulating tumor microenvironment plays a vital role in improving antitumor efficiency. Programmed Death Ligand 1 (PD-L1) is expressed in many cancer cell types, while its binding partner Programmed Death 1 (PD1) is expressed in activated T cells and antigen-presenting cells. Whereas, its dysregulation in the microenvironment is poorly understood. In the present study, we confirmed that evodiamine downregulates MUC1-C, resulting in modulating PD-L1 expression in non-small cell lung cancer (NSCLC).

**Methods:**

Cell viability was measured by MTT assays. Apoptosis, cell cycle and surface PD-L1 expression on NSCLC cells were analyzed by flow cytometry. The expression of MUC1-C and PD-L1 mRNA was measured by real time RT-PCR methods. Protein expression was examined in evodiamine-treated NSCLC cells using immunoblotting or immunofluorescence assays. The effects of evodiamine treatment on NSCLC sensitivity towards T cells were investigated using human peripheral blood mononuclear cells and Jurkat, apoptosis and IL-2 secretion assays. Female H1975 *xenograft* nude mice were used to assess the effect of evodiamine on tumorigenesis in vivo. Lewis lung carcinoma model was used to investigate the therapeutic effects of combination evodiamine and anti-PD-1 treatment.

**Results:**

We showed that evodiamine significantly inhibited growth, induced apoptosis and cell cycle arrest at G2 phase of NSCLC cells. Evodiamine suppressed IFN-γ-induced PD-L1 expression in H1975 and H1650. MUC1-C mRNA and protein expression were decreased by evodiamine in NSCLC cells as well. Evodiamine could downregulate the PD-L1 expression and diminish the apoptosis of T cells. It inhibited MUC1-C expression and potentiated CD8^+^ T cell effector function. Meanwhile, evodiamine showed good anti-tumor activity in H1975 tumor *xenograft*, which reduced tumor size. Evodiamine exhibited anti-tumor activity by elevation of CD8^+^ T cells in vivo in Lewis lung carcinoma model. Combination evodiamine and anti-PD-1 mAb treatment enhanced tumor growth control and survival of mice.

**Conclusions:**

Evodiamine can suppress NSCLC by elevating of CD8^+^ T cells and downregulating of the MUC1-C/PD-L1 axis. Our findings uncover a novel mechanism of action of evodiamine and indicate that evodiamine represents a potential targeted agent suitable to be combined with immunotherapeutic approaches to treat NSCLC cancer patients. MUC1-C overexpression is common in female, non-smoker, patients with advanced-stage adenocarcinoma.

**Supplementary Information:**

**Supplementary information** accompanies this paper at 10.1186/s13046-020-01741-5.

## Background

Lung cancer in the world is the most commonly diagnosed carcinoma and becomes a paramount cause of cancer death, which is frequently associated with poor clinical outcomes [1–3]**.**. Despite progress and development of new treatment and drugs, only 17.7% of all patients with lung cancer are alive ≥5 years after diagnosis [1, 3]. Immune cells are thought to be inactivated in tumor microenvironment, via the engagement of inhibitory receptors such as the famous Cytotoxic T-Lymphocyte Associated Protein 4(CTLA4) and Programmed cell death protein 1 (PD-1) [4–6]. Blocking immune checkpoints such as PD-L1 and PD-1 has improved the treatment of non -small cell lung cancer (NSCLC) [7]. Increasing clinical evidence shows that interaction between PD-1 and PD-L1 inhibits activation, expansion and effector functions of CD8+ T cells, and helps cancer cells evade immune destruction in the tumor microenvironment that is unfavorable to anti-cancer activities [8]. In addition, it is well known that the relation between microenvironment and immunology plays a vital role in the clinical treatment of NSCLC patients [9–11]. Activated CD8+ T cells have been demonstrated to have anti-cancer immunity in many different types of cancer [12, 13]. The presence of activated CD8+ T cells within the tumor and in the peritumoral stroma has been shown to have significant positive prognostic importance [14]. However, PD-1/PD-L1 inhibitor in clinical blockade has been limited by the response rate of about ∼20–30% in patients with NSCLC [15, 16]. The cancer microenvironment makes a vital role in the utility of immunotherapy [17–19]. Increasing clinical evidence has also shown that tumor-infiltrating CD8+ lymphocytes (TILs) in the tumor environment is associated with survival of patients with cancer, further supporting the close relationship between immune escape and tumor microenvironment [20–22].

Increasing evidence has indicated that MUC1-C plays a critical role in anti-cancer properties; for example, the transcriptional regulation of genes associated with tumor invasion [23], proliferation, metastasis [24], angiogenesis [24], apoptosis, inflammation [25], and drug resistance [26, 27] have been linked to poor outcomes in lung cancer [20]. Cancer cells usually have a high expression of MUC1-C and abnormal glycosylation of MUC1-C protein which is also overexpressed in human lung cancers and associated with a poor outcome [28]. Silencing the MUC1-C can improve the anticancer effect on lung and breast cancer [20, 28]. Accumulating evidence shows that MUC1-C regulates many genes, such as PD-L1, that promote the evasion of NSCLC cells and inhibit the effect of immune cells [29, 30]. Therefore, MUC1-C is a target for the downregulation of PD-L1 in NSCLC cells [31]. However, the effect of evodiamine on MUC1-C remains underexplored.

Evodiamine is a novel alkaloid, which was isolated from the fruit of *Tetradium* [32], and it has been considered an effective Chinese medicine for the treatment of gastropathy, hypertension, and eczema [33]. Several studies reported that evodiamine has various biological effects, including anti-nociceptive, anti-bacterial, anti-obesity and anti-cancer activities [34–36]. However, to date, the effect of evodiamine on the PD-1/PD-L1 axis remains underexplored.

In this study, the effects of evodiamine on cell viability, cell cycle, and apoptosis in the human NSCLC cell lines were investigated and the underlying mechanisms are further explored. More importantly, the anticancer and immunomodulatory activities of evodiamine in the human NSCLC cells in vitro and in vivo models are carefully examined. Our results confirm the efficacy of combining evodiamine and anti-PD-1 mAb treatment against NSCLC cells. Our research also explored the involvement of MUC1-C/PD-L1 signaling of evodiamine in anti-lung cancer. Evodiamine can improve immunity in vivo by inhibiting PD-L1 expression in cancer. Therefore, our findings disclose the inhibition of evodiamine anti-NSCLC, which might have potential clinical implications.

## Materials and method reagents

### Materials

Evodiamine was supplied by Selleck Chemicals (Houston, TX, USA) and was dissolved in dimethyl sulfoxide (DMSO) and stored at − 20 °C. Primary antibodies against GAPDH (#5174), PD-L1(#13684), MUC1-C(#16564), C-MYC(#18583) were provided by Cell Signaling Technology (Danvers, MA, USA). Fluorescein secondary antibodies were provided by LI-COR Biosciences (Lincoln, NE, USA). Dead Cell Apoptosis Kit with Annexin V-FITC/PI were provided by BD Biosciences (San Jose, CA, USA).

### Cell lines and cell culture

The proliferation of lung cancer cells was assessed using the MTT assay as described previously [37]. After treatment of 72 h, 20 μl MTT (5 mg/ml) solution was added to each well and incubated for 4 h. Then, 100 μl of the DMSO was added to each well. Finally, the colorimetric intensity of the plates was measured at the wavelength of 570 nm by the Tecan microplate reader (Morrisville, NC, USA).

### Flow cytometric analysis

Apoptosis was analysis as described previously [38]. After treatment of 24 h, the percentage of apoptotic cells on evodiamine-treated NSCLC was analyzed using a BD FACSAria III flow cytometer. The percentages of the sub-G1, S, G1 and G2 phases cells were quantitatively determined using flow cytometer. For cell surface PD-L1 on lung cancer cell lines, cells after treatment were suspended in FACS stain buffer and incubated with APC anti-human CD274 at 4 °C for 30 min and cells were resuspended, and measured analyzed using flow cytometer.

### Western blot analysis

The detailed procedure was reported previously [37]. The following antibodies were used in this experiment: GAPDH, MUC1-C (D5K9I) XP, PD-L1 and C-MYC. The protein expression was analyzed by using an LI-COR Odyssey scanner (Belfast, ME, USA).

### H1975 and H1650 co-cultured with PBMC

Human peripheral blood mononuclear cells (PBMC)/H1975 and H1650 cells were seeded at a density of 3 × 10^4^ cells. PBMCS were isolated from healthy donors by using Ficoll-Paque density centrifugation. Then, the obtained peripheral blood lymphocytes were added to the co-culture system at a ratio of 2:1. PBMC/lung cancer cell H1975 /H1650 co-cultured cells in six well plates were treated with evodiamine or vehicle. PBMC/H1975 (CshRNA), H1975 (MUC1-CshRNA) co-cultured cells and PBMC/H1650 (CshRNA), H1650(MUC1-CshRNA) co-cultured cells in 6 well plates were treated with evodiamine or vehicle. Cells were treated with evodiamine for 48 or 72 h. Afterward, lymphocyte cells were harvested from the co-culture system, and the T cells were stained for apoptosis assay.

### Transient transfection assay

Cells were transfected by using lentiviral vectors with control shRNAand MUC1-CshRNA. Puromycin was used for optimal selection of the transfected cells. For flow cytometry for cell cycle and apoptosis analysis, cells were stained with antibody in stain buffer for 30 min (in the dark at 4 °C). Cells were analyzed by BD FACSAria III flow cytometer (BD Biosciences). The surface expression of PD-L1 and MUC1-C were detected with flow cytometry.

### Protein extraction and western blotting

H1975 were treated with evodiamine for 24 h, and protein was extracted from cells by using NE-PER™ Nuclear and cytoplasm extraction reagents (Thermo Fisher Scientific, Waltham, MA, USA). The expression of PD-L1 and MUC1-C on H1975 was detected using Western blotting.

### Using quantitative real-time RT-PCR to detect the mRNA expression

After Total RNA isolated, cDNAs were synthesized by a cDNA Reverse Transcription SuperMix Kit (Bio-RAD) Using the Power SYBR Green PCR Master Mix (Roche) to detect the PD-L1 and MUC1-C mRNA expression. Primers used for qPCR were reported previously [39].

### Promoter-reporter assay

H1975 and H1650 cells were transfected with PD-L1 promoter-Luciferase reporter expression (pD-L1-Luc) or mock vehicle (Active Motif). After treated with evodiamine for 48 h, the cells were lysed and analyzed by using the Dual-Luciferase® Reporter Assay System (Promega, Madison, Wisconsin, USA).

### IL-2 assays

H1975 and H1975 MUC1-C knockout were treated with IFN-γ for 24 h. Jurkat cells were activated and added to H1975 and H1650 cells at a ratio of 2:1 or 4:1 Jurkat: H1975/H1650. The cell culture media were collected, and the IL-2 expression was measured using ELISA-kit and flow cytometry.

### Immunohistochemistry

Lung cancer patient and mouse lung cancer samples were processed for the staining of MUC1-C, PD-L1, CD4, CD8. Immunohistochemical images were captured and the numbers of MUC1-C, CD4, PD-L1, CD8, IFN-γ and Granzyme B positive cells in sections were counted.

### Tumor *xenograft* studies and Lewis lung carcinoma model

Animal experiments were performed in accordance with the guidelines by the care and use of laboratory Animals. H1975 *xenografts* on female C57BL/C mice were treatment with 10, 20, 30 mg/kg of evodiamine and *xenografts* were allowed to grow for over 1 week when tumors were detectable with calipers before treatment by gavages.

For the Lewis lung carcinoma model, the Lewis lung carcinoma cells (5 × 10^5^ cells) were intravenously injected into female C57BL/C mice at age 8–10 weeks. Single-cell suspensions of tumors and blood and spleen cells were stained, and cells were acquired using a flow cytometer and analyzed with flowJo software to detect tumor multiplicity in the lung.

### Immunohistochemistry

Human tissue of patient with NSCLCwere collected from 2014 to 2017 in Taihe Hospital. Immunohistochemistry analyses were performed using an DAKO EnVision system as described previously. The following antibodies were used: MUC1-C from Cell Signaling (catalog no. #16564). This study about patients with NSCLC was approved by the ethics committee of Hubei Taihe Hospital.

### Statistical analysis

All data in this article were expressed as the mean ± SD of three individual experiments. Differences between groups were determined by one-way analysis of variance (ANOVA) by Graph Pad Prism 8 followed by the Bonferroni test to compare all pairs of columns. When *P* < 0.05, the results were considered to be statistically significant in this study. Survival rates of mice in our experiment were generated based on the Kaplan–Meier method, statistical significance was determined by the log-rank test, *P* value < 0.05 is considered statistically significant.

## Results

### Evodiamine can inhibit growth and induce apoptosis of NSCLC cells

**Fig. 1:**
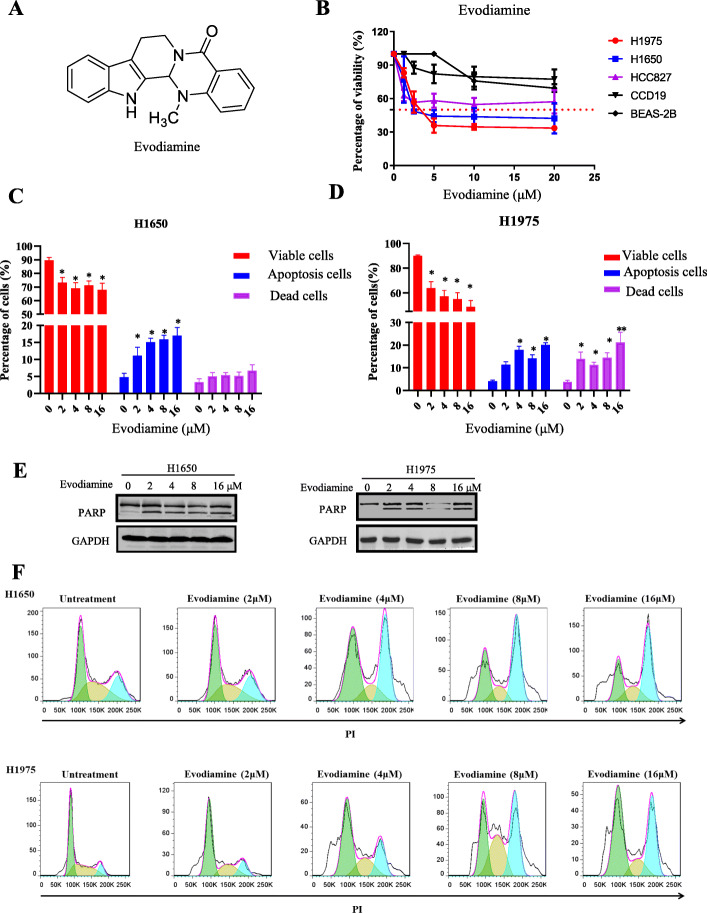
Evodiamine inhibits growth and induced apoptosis in NSCLC cells. **a** The structures of evodiamine. **b** The human Lung adenocarcinoma cell lines cells were treated with evodiamine for 72 h and cell viability was detected by MTT assays. **c-d** H1650 and H1975 cells were treated with evodiamine for 24 h and the apoptosis was analyzed by flow cytometry. **e** Cleaved PARP was also detected (**e**), and the data show that cleaved PARP was significantly increased by evodiamine treatment for 24 h. **f** H650 and H1975 cells are treated for 24 h, and cell cycle distribution is analyzed using flow cytometry. The results were presented in three independent experiments with the mean ± S.D. (*n* = 3, *P < 0.05; **P < 0.01; ***P < 0.001)

To detect the cytotoxicity of evodiamine on NSCLC cells, NSCLC cells were treated different concentration of evodiamine for 72 h. Figure 1a showed the chemical structure of evodiamine. The MTT data showed that evodiamine inhibited the growth of two cell lines (H1650 and H1975) in a dose-dependent manner (Fig. 1b). Figure 1b and Extended Data Fig. 2A show that among four cell lines (H1975, H1650, H2228 and HCC827), H1975 and H1650 are more sensitive to evodiamine, with an IC_50_ value of 5.59 ± 0.88 and 4.53 ± 1.46 μM, respectively, whereas H2228 and HCC827 were less sensitive. H1975 and H1650 are EGFR tyrosine kinase inhibitor resistant lung cancer cells. Evodiamine had much less cytotoxicity in the normal cell line CD19 and BEAS-2B (Fig. 1b and Extended Data Fig. 2A). To detect the apoptosis cells of evodiamine in H1650 and H1975 cells, the percentage of apoptosis cells was examined. As illustrated in Fig. 1c-d and Extended Data Fig. 1A-B, the proportion of apoptotic cells in H1650 and H1975 was significantly increased after treatment with evodiamine. Cleaved PARP is also detected (Fig. 1e), and the data show that cleaved PARP is significantly increased by evodiamine treatment for 24 h. The cell cycle distribution of evodiamine on H1975 and H1650shows that evodiamine inhibits H1650 and H1975 NSCLC cells, and it induces cell cycle arrest at G2 phase (Fig. 1 F and Extended Data Fig. 1B).

### Evodiamine decreases PD-L1 and MUC1-C expression in NSCLC cells

**Fig. 2:**
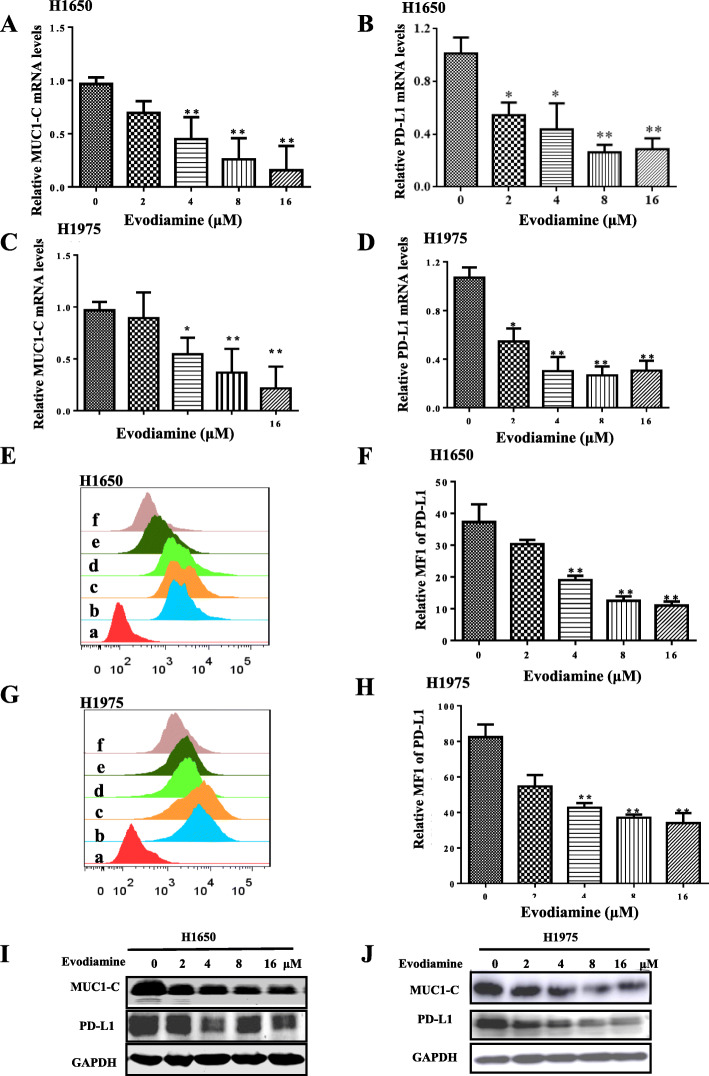
Evodiamine decreases PD-L1 and MUC1-C expression in NSCLC cells. **a-d** After H1975 and H1650 cells were treated with of evodiamine for 24 h, the mRNA expression of MUC1-C and PD-L1 is determine by quantitative RT-PCR. **e-f** H1650 and H1975 cells were treated with evodiamine for 24 h, and the membrane surface PD-L1 expression is detected by flow cytometry. **i-j** H1650 and H1975 cells are treated with different concentration of evodiamine for 24 h and western blot assays are to analyze MUC1-C and PD-L1 for 24 h. GAPDH is used as a loading control. The results are presented in three independent experiments with the mean ± S.D. (*n* = 3, *P < 0.05; **P < 0.01; ***P < 0.001). **a**: isotryposin control, **b**: control, **c**: 2 μM evodiamine, **d**:4 μM evodiamine, **e**: 8 μM evodiamine, **f**: 16 μM evodiamine

The potential signaling that may be involved in the inhibitory response by evodiamine in NSCLC cells was explored. The results show that evodiamine decreased the expression of PD-L1 mRNA and MUC1-C mRNA in H1975 and H1650 at 24 h (Fig. 2a-d). Evodiamine specifically decreases cell surface expression of PD-L1 in H1975 and H1650 cells (Fig. 2e-h) Moreover, evodiamine decreases PD-L1 and MUC1-C protein in H1975 and H1650 cells in a dose-dependent manner (Fig. 2i-j).

### Evodiamine increases cell apoptosis in NSCLC in a MUC1-C-dependent manner

**Fig. 3:**
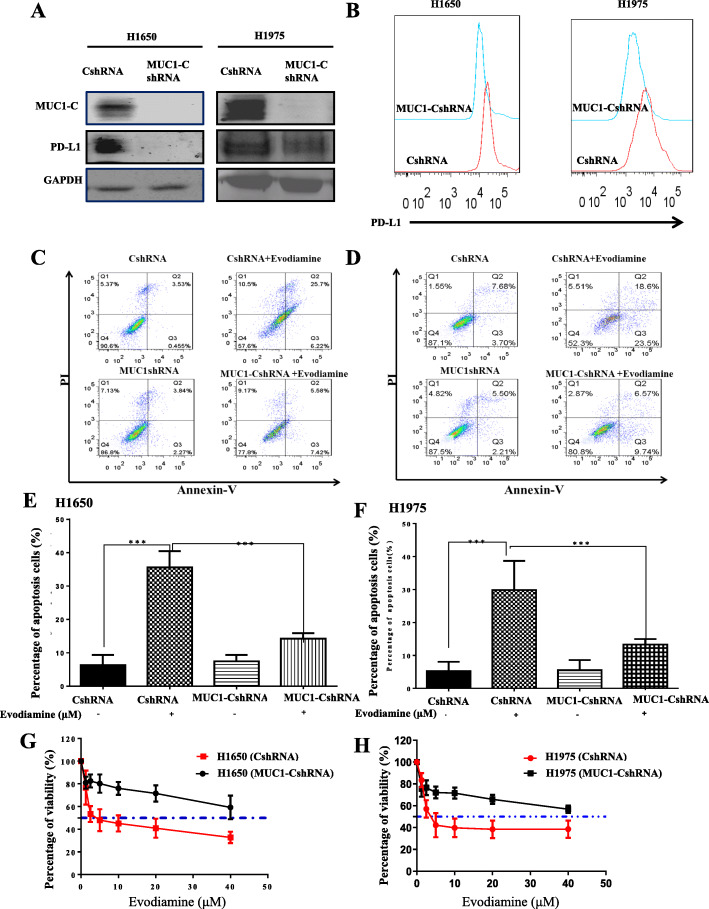
Evodiamine increases cell apoptosis in H1650 and H1975 in a MUC1-C-dependent manner. **a-b** The Western blot assays results show that MUC1-C was successfully knocked down in H1650 and H1975cells. **c-f** The percent of apoptosis cells is analyzed by using flow cytometry. **g-h** The MTT results also demonstrated that evodiamine increases cell apoptosis and inhibits growth in H1650 and H1975 via the MUC1-C dependent. The results were presented in three independent experiments with the mean ± S.D. (*n* = 3, *P < 0.05; **P < 0.01; ***P < 0.001)

The effects of targeting MUC1-C on the response of H1975 cells to evodiamine were investigated. The western blot assay results Fig. 3a demonstrate that MUC1-C was successfully knocked down in both H1975 and H1650 cell. The results of this work show that knockdown of MUC1-C in H1650 and H1975 cells results in downregulating the surface expression of PD-L1(Fig. 3b). Evodiamine-treatment induced apoptosis is significantly reduced in H1650 and H1975 with MUC1-C knockdown (Fig. 3c-f). In Fig. 3g-h), the MTT results also demonstrate that evodiamine increases cell apoptosis and inhibits growth in H1650 and H1975 in a MUC1-C dependent manner. H1975 and H1650 were transfected with control (pCMV6-AC) and MUC1-C plasmid DNA to demonstrate MUC1-C is a potential novel mechanism of evodiamine of tumor inhibition. Then cells were treated with evodiamine for an additional 24 or 72 h. Extended Data Fig. 6 data shows that expression of MUC1-C abrogates the effect of evodiamine on MUC1-C expression and cell growth inhibition. H1975 and H1650 cells were treated with evodiamine, and PD-L1 promoter activity were determined to investigate whether evodiamine activates the PD-L1 promoter. Extended Data Fig. 2B data demonstrates that evodiamine inactivates the PD-L1 promoter in H1975 and H1650.

### Evodiamine inhibits the inhibition of IFN-γ-induced PD-L1 expression in H1650 and H1975

**Fig. 4:**
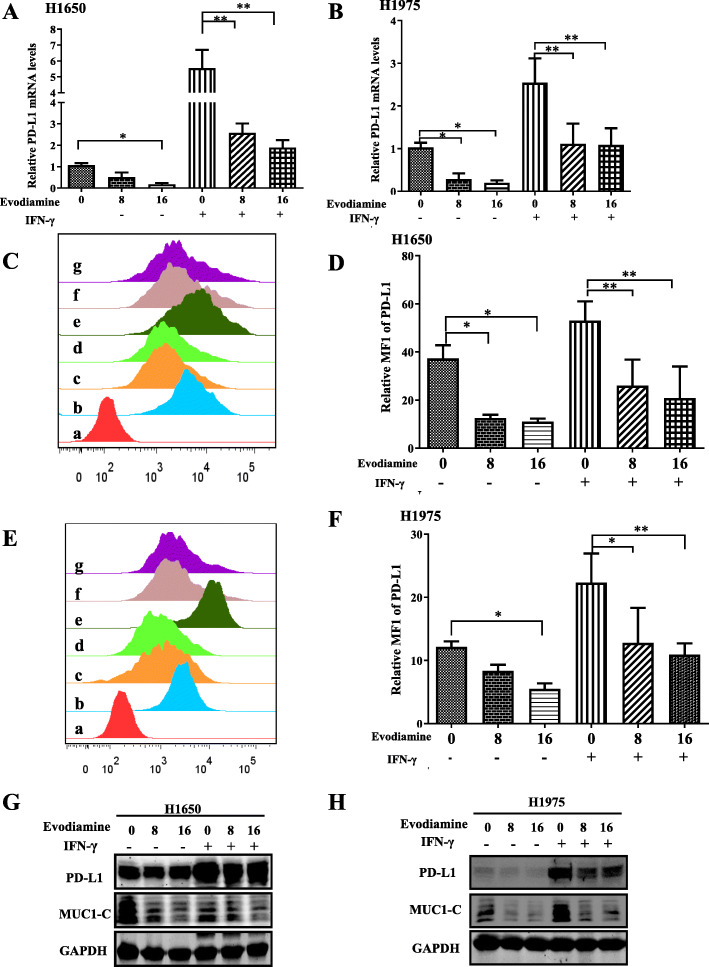
Evodiamine inhibits the inhibition of IFN-γ-induced PD-L1 expression in H1650 and H1975. **a-b** H650 and H1975 cells were pretreated with DMSO, evodiamine (8 and 16 μM) for 1 h and then treated with IFN-γ for 24 h. MUCI-1-C and PD-L1mRNA expression were determined by quantitative RT-PCR. **c-f** The cell surface PD-L1 expression on H650 and H1975 is determined using flow cytometry. The column charts below show the quantitative data of relative PD-L1 membrane protein expression. **g-h** pre-treatment of H1650 and H1975 cells with 8–16 μM evodiamine, evodiamine can decrease the IFN-γ-induced PD-L1 protein expression in H1650 and H1975 cells. The results were presented in three independent experiments with the mean ± S.D. (*n* = 3,*P < 0.05; **P < 0.01; ***P < 0.001) a: isotryposin control, b: control, c: 8 μM evodiamine, d:16 μM evodiamine, e: IFN-γ (10 ng/ml), f: 8 μM evodiamine +IFN-γ (10 ng/ml), g: 16 μM evodiamine++IFN-γ (10 ng/ml)

Interferon-γ (IFN-γ) is an important cytokine with different pleiotropic in the tumor microenvironment, has long been praised as an effector cytokine of anti-cancer immunity and can inhibit cancer development and growth through various mechanisms. However, IFN-γ has also been involved in tumor cell development and transformation. IFN-γ also helps cancer cells evade immune destruction, suggesting that IFN-γ has an existing side effect of promoting tumor. Reports showed that IFN-γcan stimulate the expression of PD-L1 in tumor cells, which bind with PD-1, inhibiting activation, expansion, and effector functions of CD8^+^ T cells, and helping cancer cells evade immune destruction to contribute to tumor immune evasion [40]. To effect of evodiamine on the IFN-γ-induced upregulation of PD-L1 expression in H1650 and H1975 cells was assessed. The optimal induction of PD-L1 was obtained after 24 h treatment with 50 ng/ml IFN-γ, Pretreatment of H1650 cells with 8–16 μM evodiamine inhibited IFN-γ-induced PD-L1 and MUC1-C mRNA expression (Fig. 4a). The experiment was repeated with H1975 that also showed inducible expression PD-L1 as well as H1650 cells that constitutively expressed PD-L1 (Fig. 4b) to confirm that the effect of evodiamine on PD-L1 expression is not cell line specific. PD-L1 expression on the cell membrane plays an important role in tumor escape. The interaction between PD-1 on T cells and PD-L1 on tumor cells on cell membrane inhibits activation, expansion, and effector functions of CD8^+^ T cells and helps cancer cells evade immune destruction. Remarkably, overexpression of PD-L1 in human cancers is associated with poor clinical outcomes. Thus, flow cytometry was used to examine the surface expression of PD-L1 on H1650 and H1975 cells after evodiamine treatment, and flow data show that evodiamine specifically decreases IFN-γ-induced surface expression of PD-L1 in H650 and H1975 (Fig. 4c-f). Moreover, pre-treatment of H1650 cell lines cells with 8–16 μM evodiamine can decrease the IFN-γ-induced PD-L1 protein expression in H1650 and H1975 cells (Fig. 4g-h).

### MUC1-C inhibition could diminish the PD-L1 expression to decrease the T cell apoptosis

PBMC cells were co-cultured with H1650 or H1975 cells, both expressing high PD-L1. The results show that the apoptosis rate of T cells increased (Extended Data Fig. 3). Extended Data Fig. 3A-D show that evodiamine can elevate CD8^+^ T cell function by increasing IFN-γ and GzmB expression. MUC1-C expression was silenced with shRNA in H1975 and H1650 cells to understand whether MUC1-C could affect T cells proliferation and apoptosis when they were co-cultured with H1975 and H1650 cells. Extended Data Fig. 3E-F showthat the apoptosis of T cells in co-culture assay decreased. These results indicate that inhibiting MUC1-C in H1650 and H1975 could reverse the apoptosis of T cells. Evodiamine, which inhibits MUC1-C in H1650 and H1975, also reversed the apoptosis of T cells. We hypothesize that evodiamine downregulates PD-L1 expression in H1975 and H1650 by targeting the MUC1-C. In our T cell-mediated lung cancer cell killing assays, evodiamine-treated H650 and H1975 cells displayed enhanced sensitivity towards activated Jurkat T cells. In our study, evodiamine could significantly increase of IL-2 secreted detected from co-cultures (Extended Data Fig. 2C).

### Evodiamine inhibits H1975 tumor *xenograft* tumor growth by downregulation of MUC1-C

**Fig. 5:**
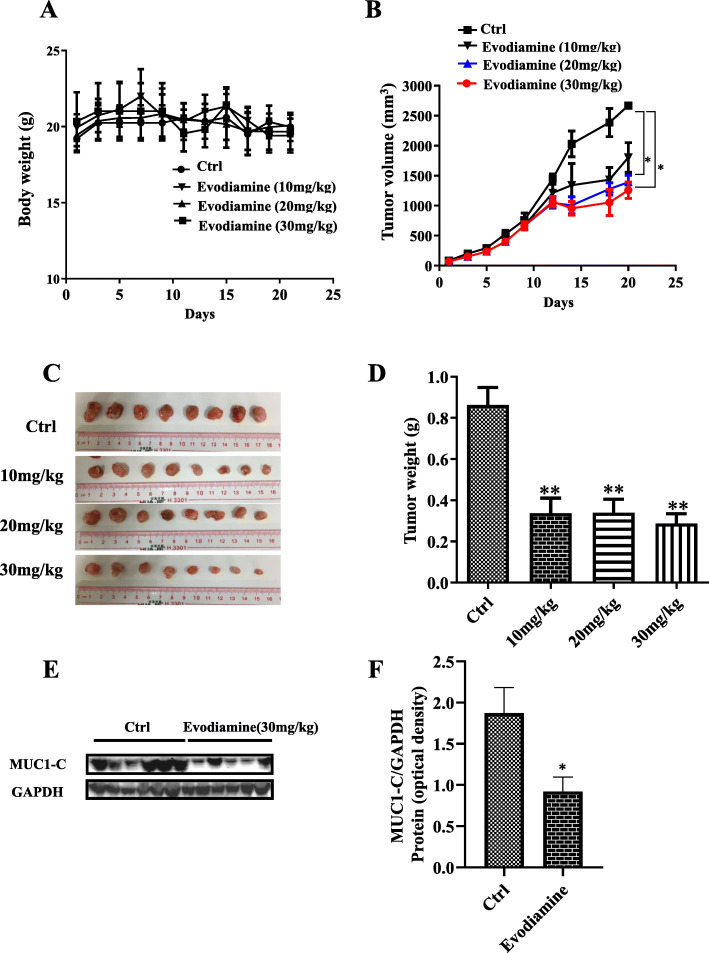
Evodiamine inhibits H1975 tumor *xenografts* tumor growth by downregulation of MUC1-**C**. **a** The body weight of evodiamine-treated mice does not change compared to untreated animals. **b-d** Verifying the effect of evodiamine in vivo, shows that compared with the controls, tumor growth exhibits a suppressive trend starting at day 15 in the evodiamine-treated mice. **e** and **f** Western blot analysis shows that MUC1-C downregulation after evodiamine treatment

H1975 xenograft was performed on female nude mice in this experiment to verify the effect of evodiamine in vivo. The body weight of evodiamine-treated mice did not change when compared to untreated animals (Fig. 5a). Compared with the controls, tumor growth control showed a suppressive trend starting at day 15 in the evodiamine-treated mice, with smaller tumor volume (Fig. 5b), size (Fig. 5c) and weight (Fig. 5d). The western blot analysis in Fig. 5e shows that MUC1-C downregulation after evodiamine treatment. Consistent with novel discovery in vitro ((Fig. 5f)), the levels of MUC1-C were all downregulated by evodiamine treatment. These data altogether show that evodiamine can inhibit NSCLC growth in vivo and in vitro at least partially by downregulation of MUC1-C.

### Evodiamine exhibits anti-tumor activity by elevation of CD8+ T cells in vivo

**Fig. 6:**
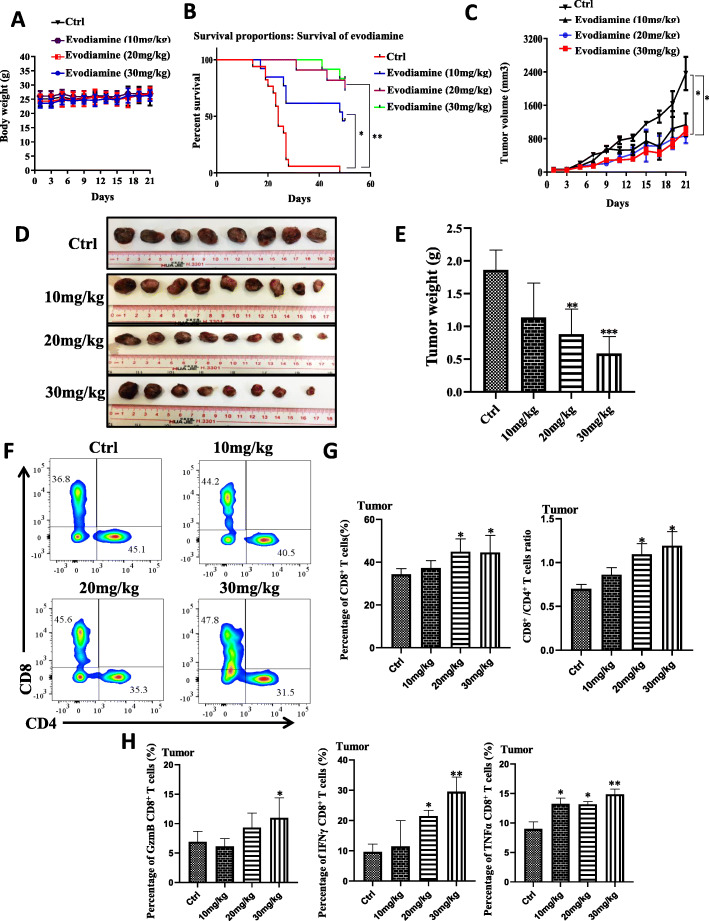
Evodiamine exhibits anti-tumor activity by elevation of CD8+ T cells in vivo. **a** Evodiamine treatment does not change the body weight. **b** The survival rate of LLC and evodiamine improves the survival of the mice. **c**-**e** Tumor volume is checked at the beginning of the treatment period. Evodiamine-inhibited repressed tumor growth is forecast to estimate the tumor volumes (**c**) and tumor size (**d** and **e**). **f**-**h**. Lymphocytes cells of tumor preparations are analyzed using flow cytometry with TNFα and Granzyme B, FIN-γ, CD4 and CD8, antibodies. The results were presented in three independent experiments with the mean ± S.D. (*n* = 3, *P < 0.05; **P < 0.01; ***P < 0.001)

The Lewis lung carcinoma model was used to study the activity of evodiamine on CD8^+^ T cells in controlling tumor progression and metastasis to verify the effect of cancer immunotherapies in mice with evodiamine. The body weight of evodiamine-treated mice did not change compared to untreated animals (Fig. 6a).

Evodiamine improved the survival of the mice (Fig. 6b). Compared with the controls, tumor growth control showed a suppressive trend starting at day 18 in the evodiamine-treated mice (Fig. 6c) and have small volume (Fig. 6c) and size (Fig. 6d) and weight (Fig. 6e). The tumor-infiltrating T cells in treatment group were analyzed, and the CD8^+^ T cells had better activity and increased cell numbers compared with the control group (Fig. 6f-g). In the lung mice model, the CD8^+^ T cells of the evodiamine treatment mice had higher activity than those of control mice (Fig. 6f-h, Extended Data Fig. 4). Evodiamine could enhance the effector function of mouse CD8^+^ T cells in vivo. The number of tumor-infiltrating CD8^+^ T cells in evodiamine-treated mice increased (Fig. 6f-g, Extended Data Fig. 4L), and these cells showed potentiated effector function.

### Combination evodiamine and PD-1 mAb treatment enhanced tumor growth control and survival of Lewis lung carcinoma model

**Fig. 7:**
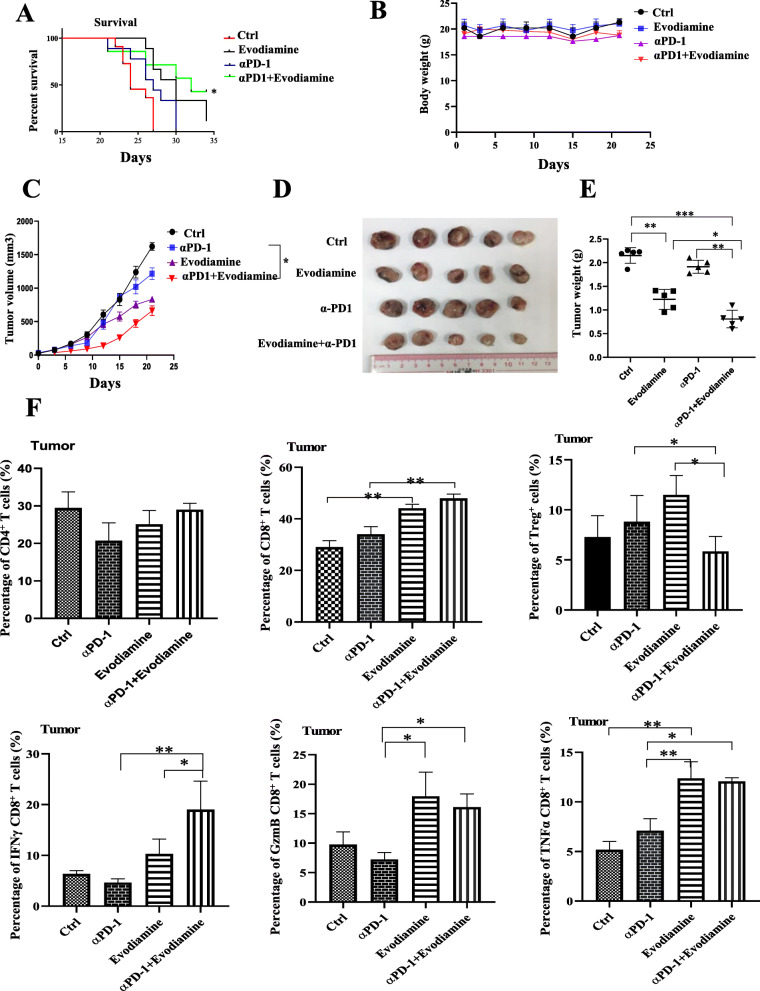
Combination of evodiamine and PD-1 mAb treatment enhance tumor growth control and survival of the Lewis lung carcinoma model. **a** The survival rate of LLC and the combination of evodiamine and PD-1 mAb improves the survival of the mice. **b** The body weight of mice does not change. Tumor volume is checked at the beginning of the treatment period. The combination evodiamine and PD-1 mAb treatment enhanced inhibits tumor growth in the Lewis lung carcinoma model (**c-e**), The efficacy of anti-tumor growth is estimated by measuring tumor volume (**c**), tumor size (**d**) and tumor weight (**e**). **h** Lymphocytes cells are analyzed using flow cytometry with FIN-γ, TNFα and Granzyme B, CD4 and CD8 antibodies. The results were presented in three independent experiments with the mean ± S.D. (*n* = 3, *P < 0.05; **P < 0.01; ***P < 0.001)

Next, the possibility of combination evodiamine and PD-1 mAb to improve the therapeutic effects of either drug alone was investigated. Treatment with combination evodiamine and PD-1 mAb improve the survival of the mice (Fig. 7a). The body weight of evodiamine-treated mice did not change compared to untreated animals (Fig. 7b). The results show that a single PD-1 mAb treatment failed to restrain tumor growth in the mouse model effectively (Fig. 7c-e), and mice treated with a combination of evodiamine and PD-1 mAb had small volume (Fig. 7c) and size (Fig. 7d) and weight (Fig. 7e). These data revealed that the percentage of CD4^+^ T cells in blood, spleen or tumor is largely unchanged by either evodiamine or anti-PD-1 treatment or the combination (Extended Data Fig. 5). By contrast, we found significantly more tumor-infiltrating CD8^+^ T cells measured by frequency and number in the combination PD-1 antibody and evodiamine treatment group (Fig. 7f). We also found that in the combination PD-1 antibody and evodiamine treatment group, tumor-infiltrating CD8^+^ T cells showed stronger activation phenotypes and more increased IFN-γ, TNFα and Granzyme B (GrzmB) production (Fig. 7f). Extended Data Fig. 5C shows the proportion of regulatory T (Treg) cells in combination evodiamine and PD-1 mAb treatment (CD4^+^FoxP3^+^T cell) in the spleen, blood and tumor is decreased (Fig. 6f and Extended Data Fig. 5).

### MUC1-C expression is highly expressed in NSCLC, have high positive rates in female, non-smoker, adenocarcinoma and advanced stage

Finally, the clinical relevance of MUC1-C in tumor of patient was investigated, and MUC1-C expression in 131 of patients with NSCLC was detected using immunohistochemical method. MUC1-C was higher in patients with late-stage (III and IV) than in the patients with early-stage (I) tumors (Table. 1). A high level of MUC1-C is associated with advanced lung cancer progression. These results indicated that MUC1-C is highly expressed in NSCLC, has high positive rates in female, non-smoker, adenocarcinoma and advanced-stage patients. Compared with healthy donors, NSCLC tumor tissue has high PD-L1 and MUC1-C mRNA expression (Extended Data Fig. 2D-E).

**Table 1:** Clinical pathological characteristics correlation analysis with MUC1-C expression in a 131 NSCLC cohort

Characteristics	Total	MUC1-C expression			F/X^**2**^	***P***-value
		Negative (%)	Low (%)	High (%)		
**Age (years)**					0.776	0.678
< =65	105	7 (6.7)	32 (30.5)	66 (62.9)		
> 65	26	1 (3.8)	10 (38.5)	15 (57.7)		
**Gender**					16.215	< 0.001
Male	105	8 (7.6)	41 (39.0)	56 (53.3)		
Female	26	0 (0.0)	1 (3.8)	25 (96.2)		
**Smoking history**					17.501	< 0.001
Yes	90	8 (8.9)	37 (41.1)	45 (50.0)		
No	41	0 (0.0)	5 (12.2)	36 (87.8)		
**Pathological pattern**					60.153	< 0.001
Adenocarcinoma	74	0 (0.0)	7 (9.5)	67 (90.5)		
Squamous cell	57	8 (14.0)	35 (61.4)	14 (24.6)		
**Cell differentiation**					5.542	0.236
Poorly	28	1 (3.6)	12 (42.9)	15 (53.6)		
Moderately	93	5 (5.4)	28 (30.1)	60 (64.5)		
Well	10	2 (20.0)	2 (20.0)	6 (60.0)		
**TNM stage**					13.306	0.038
I	62	4 (6.5)	18 (29.0)	40 (64.5)		
II	37	3 (8.1)	19 (51.4)	15 (40.5)		
III	28	1 (3.6)	5 (17.9)	22 (78.6)		
IV	4	0 (0.0)	0 (0.0)	4 (100.0)		

## Discussion

In the present study paper, we demonstrated that evodiamine not only significantly inhibits proliferation and increases apoptotic but also shows good anti-tumor activity, which reduces tumor size in H1975 tumor *xenograft* and Lewis lung carcinoma model,. We demonstrated that knock out the MUC1-C in H1650 and H1975, both expressing high MUC1-C, can increase IC_50_ of evodiamine effected on these two cell lines. In our experiment, we observed the inhibitory effect of PD-L1 mRNA and membrane PD-L1 following treatment with evodiamine in IFN-γ stimulation H1650 and H1975. Activation PD-L1 could, through the PD-L1/PD-1 axis could induce the T cells apoptosis in the co-culture system. Evodiamine could downregulate the PD-L1 expression to diminish the apoptosis of T cells. It inhibited MUC1-C expression and potentiated CD8^+^ T-cell effector function. Evodiamine exhibited anti-tumor activity by elevating of CD8^+^ T cells in vivo in the Lewis lung carcinoma model. We observed that combination of evodiamine and anti-PD-1 mAb treatment enhanced tumor growth control and survival.

The potential impact of Chinese herbal medicines and their ingredients in numerous types of tumor treatment has attracted widespread attention [41]. Extensive investigations demonstrated that evodiamine, an alkaloid extracted from *Euodia rutaecarpa*, inhibit the proliferation of various tumors and induce tumor cell apoptosis [42]. Evodiamine isthe one of the most popular, multi-purpose Chinese herbal medicine for the treatment of many indications, including headaches, menstruation disorder, amenorrhea, abdominal, pain, diarrhea, vomiting, postpartum hemorrhage, gastrointestinal disorders and others [43–45]. Evodiamine, as a novel occurring indole alkaloid with attractive multi-targeting antiproliferative activity, has been investigated as a leading compound that possesses multi-targeting profiles [43, 44, 46]. Evodiamine derivatives with Novel boron-containing were designed, which have improved the anti-tumor potency of the evodiamine and showed a good antitumor activity in vitro and in vivo by reactive oxygen species (ROS) [43]. More and more evidence have reported that evodiamine has inhibitory effects on lung tumor growth and metastasis by suppressed cell viability, induced G2/M cell cycle arrest and inhibited cell migration [45]. However, the anti-cancer mechanisms of evodiamine on the immune checkpoint PD-1/PD-L1 axis and its immune effects remain underexplored until now. We confirmed that in the current study evodiamine increases cell apoptosis in NSCLC in a MUC1-C-dependent manner. Evodiamine has a good anti-cancer tumor function by inhibiting NF-κB [47, 48]. A Bouillez reported that MUC1-C can drives the transcription of CD274 in tumor cells through NF-κBp65 or C-MYC on the PD-L1 promoter, resulting in integrating PD-L1 activation on cancer cells with suppression of immune effectors on T cells and then helping cancer cells immune evasion and poor clinical outcome [31, 39]. Our data demonstrated that evodiamine could downregulate MUC1-C and decrease the PD-L1 promoter in H1975 and H1650 cells (Extended Data Fig. 2B and Extended Data Fig. 6), leading to inhibiting MUC1-C expression and potentiating CD8^+^ T-cell effector function.

Tumor vaccine fused with soluble PD-1 with the MUC1 gene showed good immunogenicity and anti-tumor effect by enhancing the activation of lymphocytes, and accumulates CD8^+^ tumor-infiltrating lymphocytes, resulting in reduced in tumor growth [49, 50]. MUC1 has been considered a possible immunotherapeutic target. TG4010, a therapeutic cancer vaccine, expressing MUC1 as well as interleukin 2, and is combine with PD-L1 immune inhibitor nivolumab, has been approved by the FDA for the first-line treatment of patients with NSCLC [51]. Chimeric antigen receptors (CARs) targeting T cells to MUC1 were developed and demonstrated the therapeutic efficacy of CAR T cells directed against Tn-MUC1 and presented aberrantly glycosylated antigens as a clinical trial to target solid cancers which express MUC1 with CAR T cells [52–54]. Consistently, Xiuling Xu reported that knockdown of MUC1-C expression in A549 and H460 effectively increases the sensitivity of these cells to the apoptotic cytotoxicity of anti-cancer therapeutics, suggesting that MUC1-C may contribute to acquired chemoresistance [28]. IFN-γ, an important cytokine in tumor microenvironments, is secreted from various types of immune cells such as T cells, activate macrophages, and B cells and natural killer (NK) cells. IFN-γ could potentially increase the expression of PD-L1 in tumor cells, which biding with PD-1, in resulting inhibiting activation, expansion, and effector functions of CD8+ T cells and helps cancer cells evade immune destruction so that to contribute to tumor immune evasion. Similarly, in our T cell-mediated lung cancer cell killing assays, evodiamine-treated H650 and H1975 cells displayed enhanced sensitivity towards activated Jurkat T cells. In our study, evodiamine could block the interaction of the PD-1/PD-L1 axis, resulting in a significant increase of IL-2 secreted detected from co-cultures. In addition to its impact on cell membrane signaling, MUC1-C is imported to the nucleus, where it associates with a different transcription factor, including p53, NF-κB p65, STAT1/3, c-MYC, HIF-1 among others. Xiuling Xu reported that knockdown of MUC1-C expression in A549 and H460 effectively increased the sensitivity of these cells to the apoptotic cytotoxicity of anti-cancer therapeutics, suggesting that MUC1-C may contribute to acquired chemoresistance. In our study, we found that silencing the MUC1-C in H1975 and H1650, both expressing high MUC1-C, can attenuate the cytotoxicity effect of Evodiamine. MUC1-C is a transmembrane subunit of the MUC1 glycoprotein, which can translocate into the nucleus and trigger expression of other cancer-related oncogenes. Interestingly, we also observed an inhibitory effect of Evodiamine on the expression of PD-L1 mRNA and membrane PD-L1 following IFN-γ stimulation. Similarly, we found that evodiamine inhibits the translocation of MUC1-C to the nucleus and suppress PD-L1 expression as well in the nuclei of NSCLC cells with IFN-γ-caused PD-L1 expression [31]. Evodiamine inhibits MUC1-C expression in the nucleus, where it associates with various transcription factor [55].

MUC1-C is a transmembrane glycoprotein, which is aberrantly expressed in > 80% Stage IB NSCLC [31, 55, 56]. Our IHC results are incompatible with those mentioned above. It should be noted that stage IB lung carcinomas were involved in Dongrong Situ et al.’s study [31]. By contrasts, our study focused on population of patients with I–IV lung carcinomas diseases, including adenocarcinoma and Squamous Cell Cancer (Table.1). We found the MUC1-C expression has high positive rates in female, non-smoker patients with advanced stage patients. Here, We reported a novel finding that evodiamine inhibits PD-L1 expression in lung adenocarcinoma by targeting MUC1-C. Suppression of MUC1-C expression via MUC1-C shRNA resulted in a decrease of PD-L1 protein and mRNA expression [57]. Evodiamine can also decrease MUC1-C and PD-L1 protein and mRNA levels in NSCLC through inhibiting MUC1-C expression. Silencing MUC1 is associated with a C-MYC decrease of PD-L1 mRNA expression, suggesting that MUC1-C regulation of PD-L1 is likely mediated by transcriptional mechanism [31]. GO203, targeting MUC1-C, can suppress PD-L1 expression of NSCLC and breast cancer, and induces effectors of innate and adaptive immunity, resulting in improve anticancer effects [31] We determined that evodiamine can block PD-L1 expression on mRNA and protein levels, perhaps through inhibiting the MUC1-C. Silencing MUC1-C in H1975 decreases apoptosis following evodiamine treatment. Currently, several preclinical and clinical trials for the combination of other therapy with immune inhibitors in the treatment patients reveal biomarkers of response and resistance to anti-PD-1 monotherapy and combined anti-CTLA-4 and anti-PD-1 immunotherapy, significantly improve the anti-tumor effect [6, 58–61]. Collectively, the combination of evodiamine and PD-1 mAb treatment enhance anti-cancer and survival in a Lewis lung carcinoma model.

## Conclusions

Overall, our results show that evodiamine suppresses NSCLC by elevation of CD8^+^ T cells and downregulation of the MUC1-C/PD-L1 axis. This paper corroborates a potential novel mechanism of evodiamine that controls Lung adenocarcinoma cell growth. Our data implies that the rational targeting MUC1-C combined with immunotherapeutic approaches is a potential therapeutic strategy against lung cancer. In our clinical pathological characteristic correlation study, the result indicated that MUC1-C is highly expressed in NSCLC, has high positive rates in female, non-smoker patients with advanced-stage adenocarcinoma. Blocking the MUC1-C/PDL-1 axis is a potential new therapeutic strategy for these subgroup of advanced stage patients.

## Supplementary Information

**Additional file 1: Extended Data Figure S1.** Evodiamine can inhibit growth and induce apoptosis of NSCLC cells. **Extended Data Figure S2.** Evodiamine inactivates the PD-L1 promoter. **Extended Data Figure S3.** MUC1-C inhibition can diminish the PD-L1 expression and decrease the apoptosis levels of CD8+T cells. **Extended Data Figure S4.** Evodiamine potentiates the anti-tumor activity of CD8+ T cells *in vivo.* **Extended Data Figure S5.** Combination Evodiamine and PD-1 mAb treatment can enhance tumor growth control and survival of Lewis lung carcinoma model. **Extended Data Figure S6.** MUC1-C is a potential novel mechanism of evodiamine of tumor inhibition.

## Data Availability

All data generated or analyzed during this study are included in this published article.

## Abbreviations

NSCLC: Non-small cell lung cancer
TCM: Traditional Chinese medicine
MUC1: MUCIN 1, cell surface associated
PD-L1: Programmed Death Ligand 1
PBMC: Human peripheral blood mononuclear cells
EGFE: Epidermal growth factor receptor
CTLA4: Cytotoxic T-lymphocyte associated protein 4
PBS: Public Broadcasting Service
PD-1: Programmed cell death protein 1
