# Supplementary material for: Evodiamine suppresses non-small cell lung cancer by elevating CD8+ T cells and downregulating the MUC1-C/PD-L1 axis
Source: J Exp Clin Cancer Res. 2020 Nov 19;39:249. doi: 10.1186/s13046-020-01741-5 (PMC7677782; doi:10.1186/s13046-020-01741-5)
Supplement: Supplementary file 1 — Additional file 1: Extended Data Figure S1. Evodiamine can inhibit growth and induce apoptosis of NSCLC cells. Extended Data Figure S2. Evodiamine inactivates the PD-L1 promoter. Extended Data Figure S3. MUC1-C inhibition can diminish the PD-L1 expression and decrease the apoptosis levels of CD8+T cells. Extended Data Figure S4. Evodiamine potentiates the anti-tumor activity of CD8+ T cells in vivo. Extended Data Figure S5. Combination Evodiamine and PD-1 mAb treatment can enhance tumor growth control and survival of Lewis lung carcinoma model. Extended Data Figure S6. MUC1-C is a potential novel mechanism of evodiamine of tumor inhibition. [file 13046_2020_1741_MOESM1_ESM.pptx]

## Slide 1
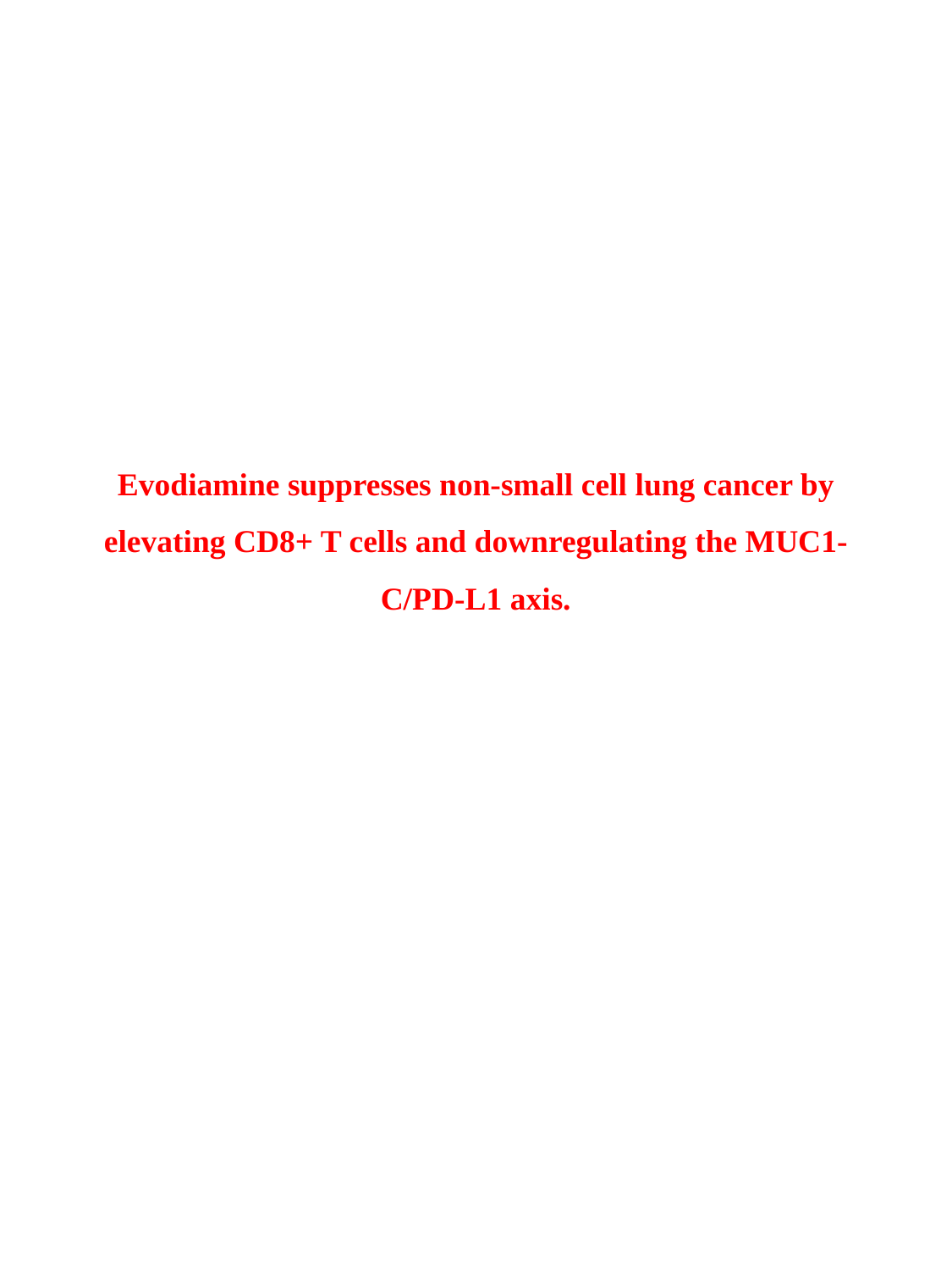

# Evodiamine suppresses non-small cell lung cancer by elevating CD8+ T cells and downregulating the MUC1-C/PD-L1 axis.

## Slide 2
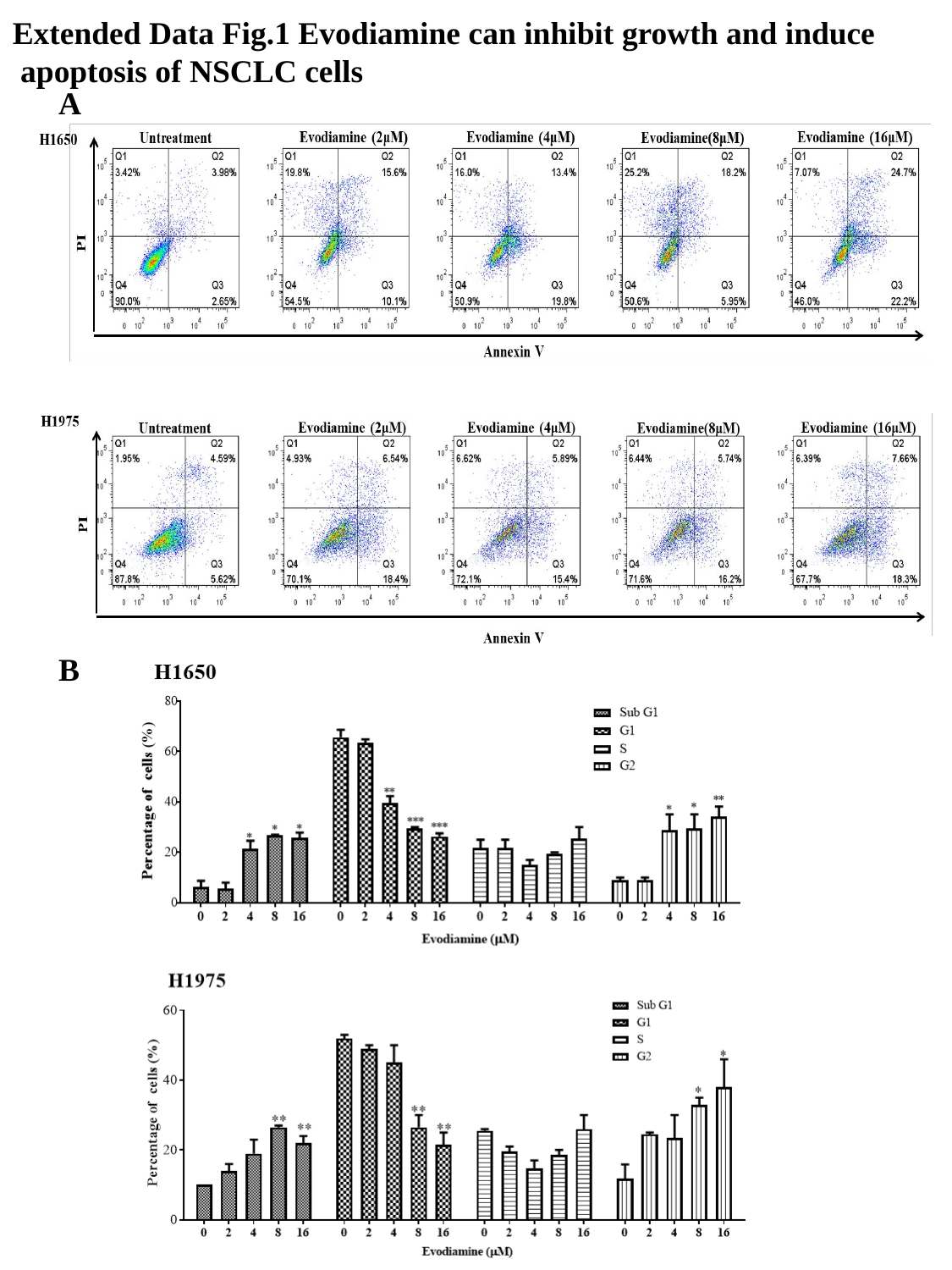

Extended Data Fig.1 Evodiamine can inhibit growth and induce
 apoptosis of NSCLC cells
A
B

## Slide 3
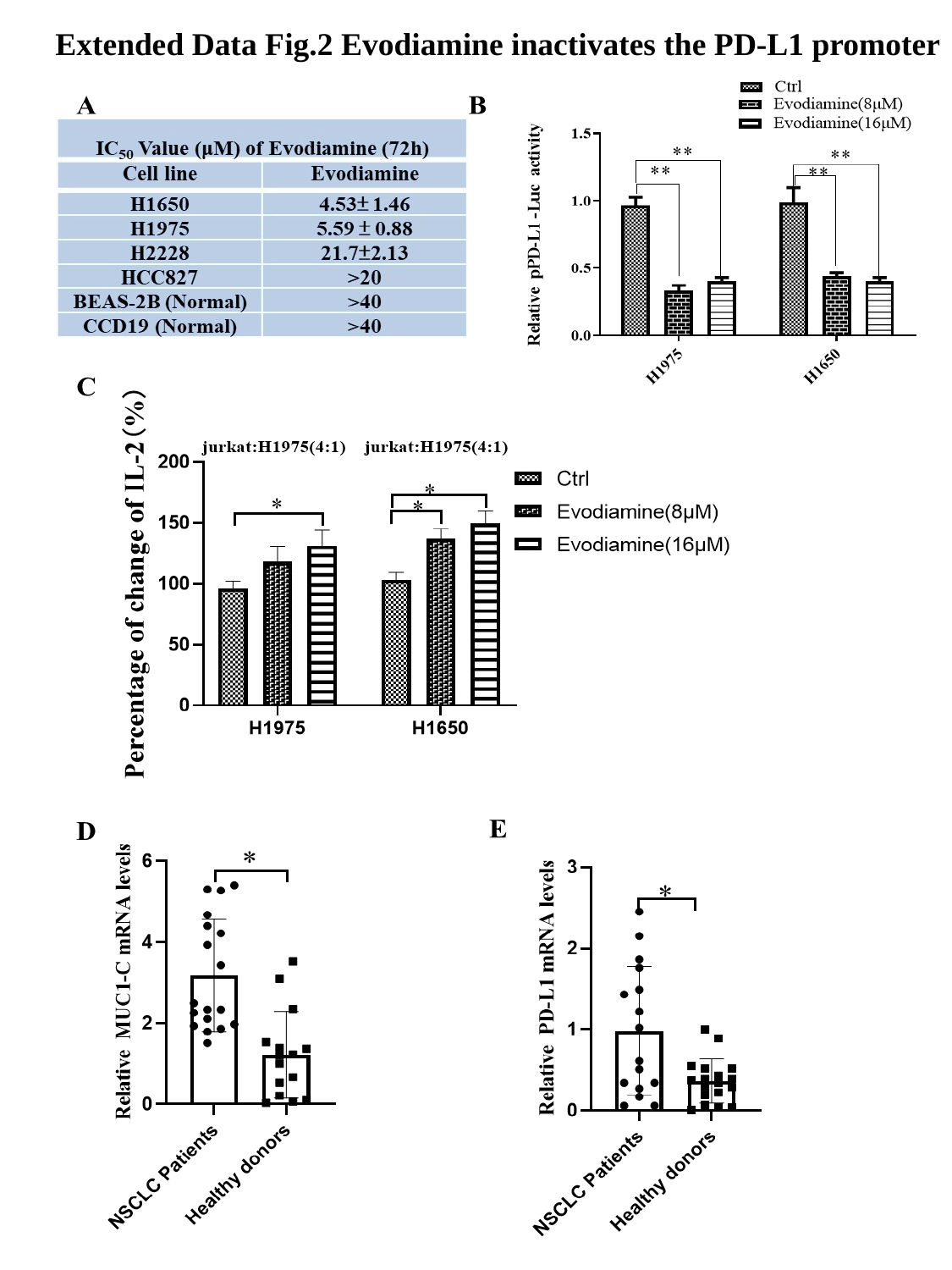

Extended Data Fig.2 Evodiamine inactivates the PD-L1 promoter

## Slide 4
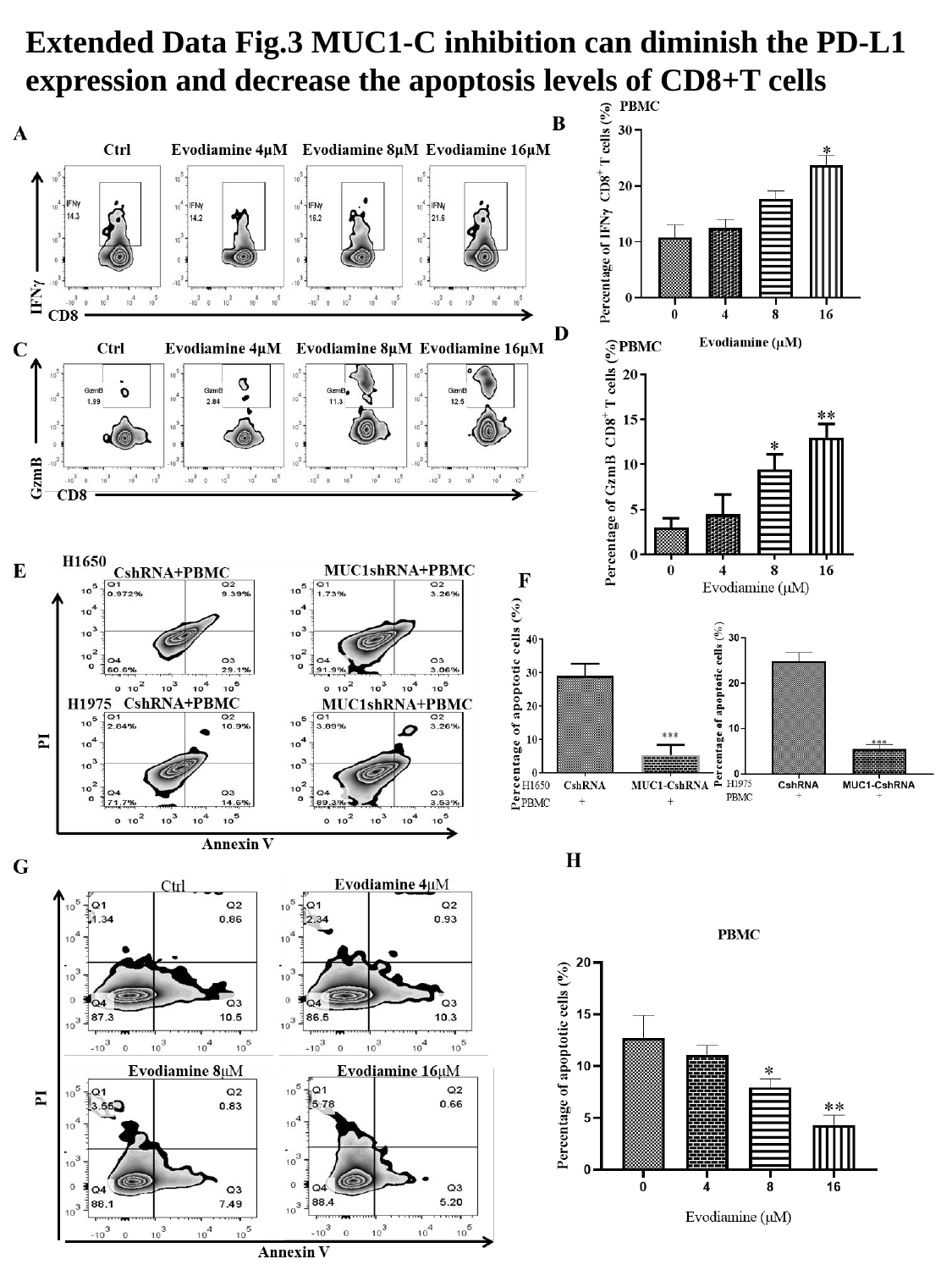

Extended Data Fig.3 MUC1-C inhibition can diminish the PD-L1 expression and decrease the apoptosis levels of CD8+T cells

## Slide 5
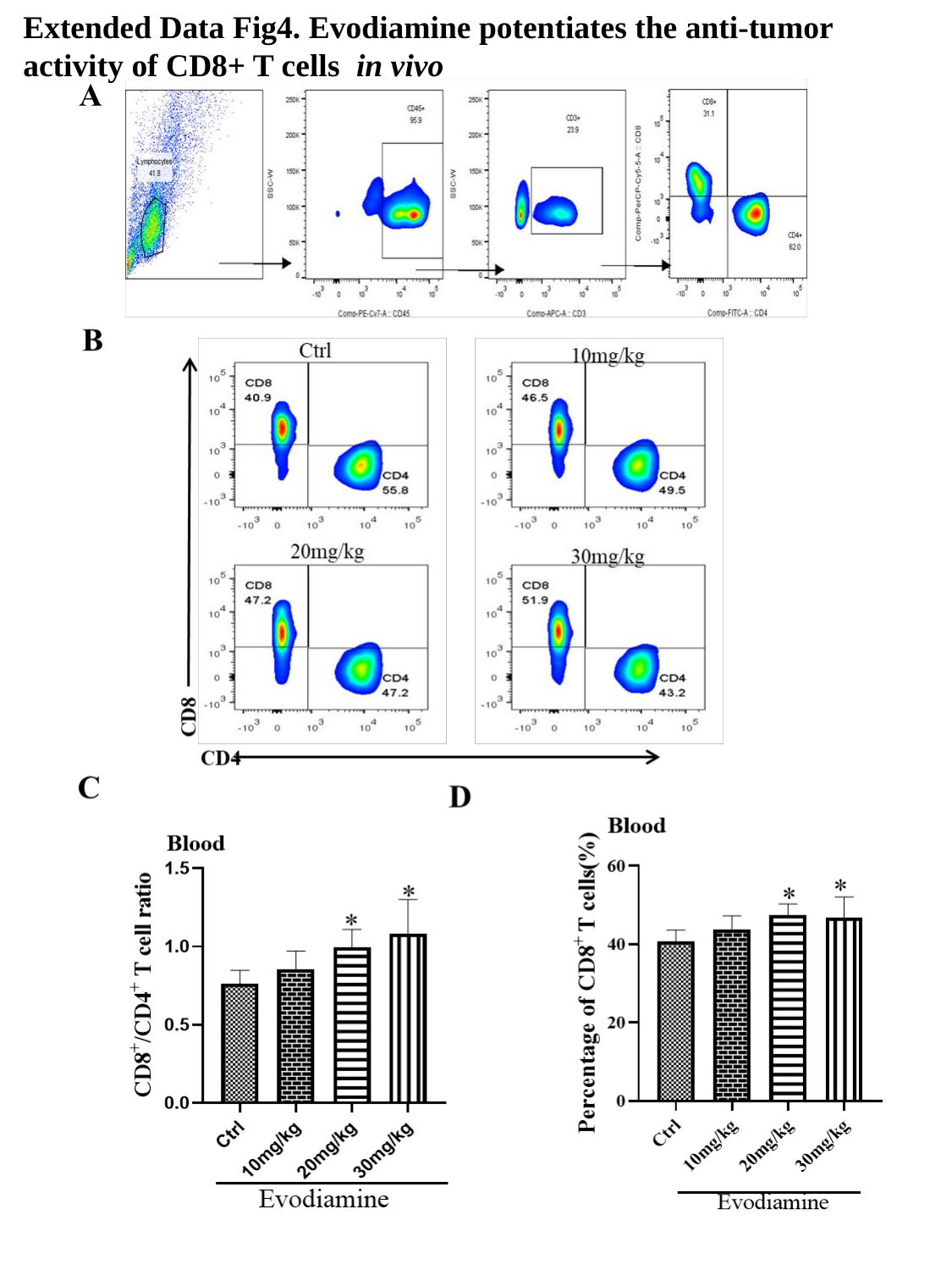

Extended Data Fig4. Evodiamine potentiates the anti-tumor
activity of CD8+ T cells in vivo

## Slide 6
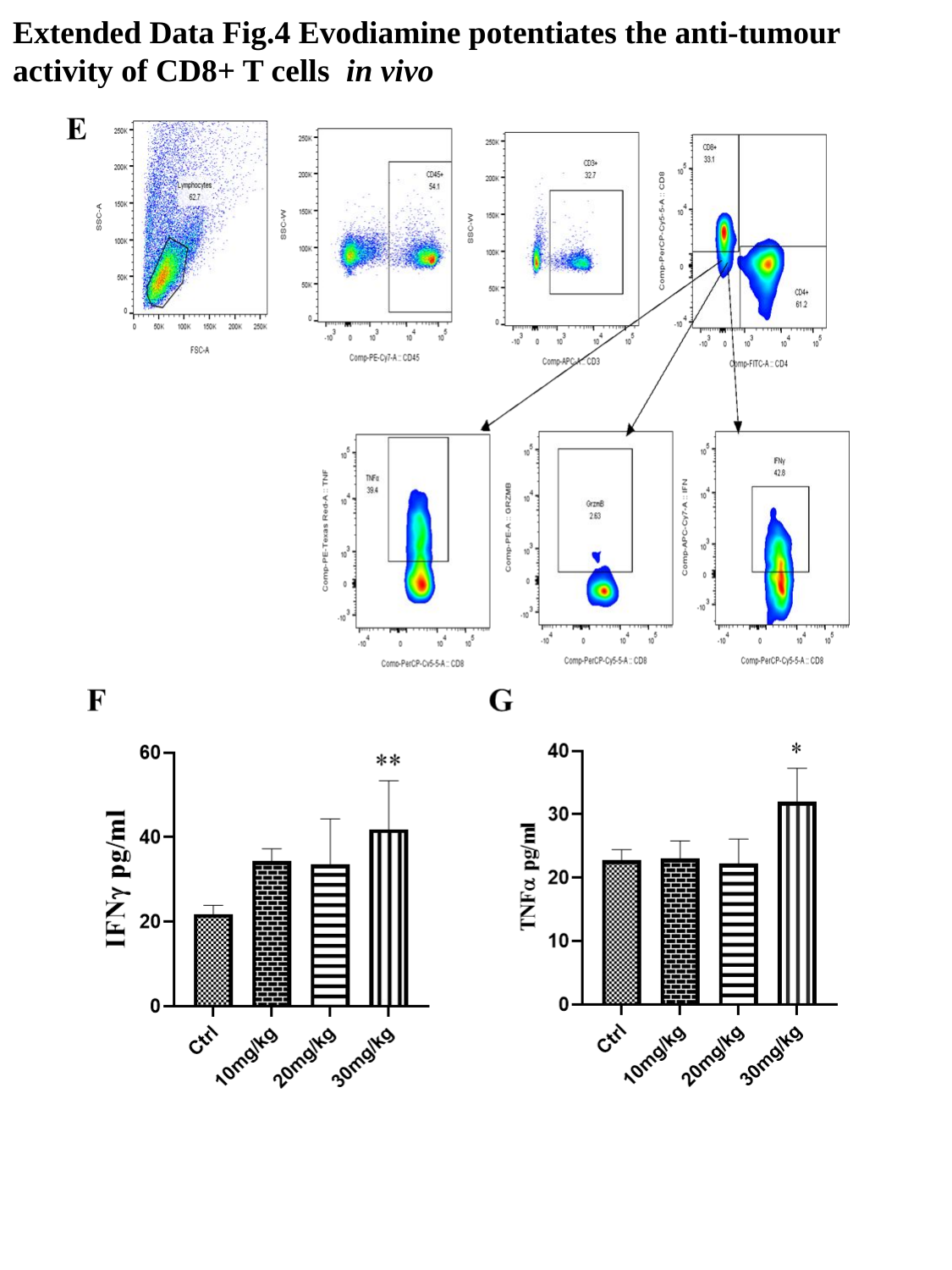

Extended Data Fig.4 Evodiamine potentiates the anti-tumour
activity of CD8+ T cells in vivo

## Slide 7
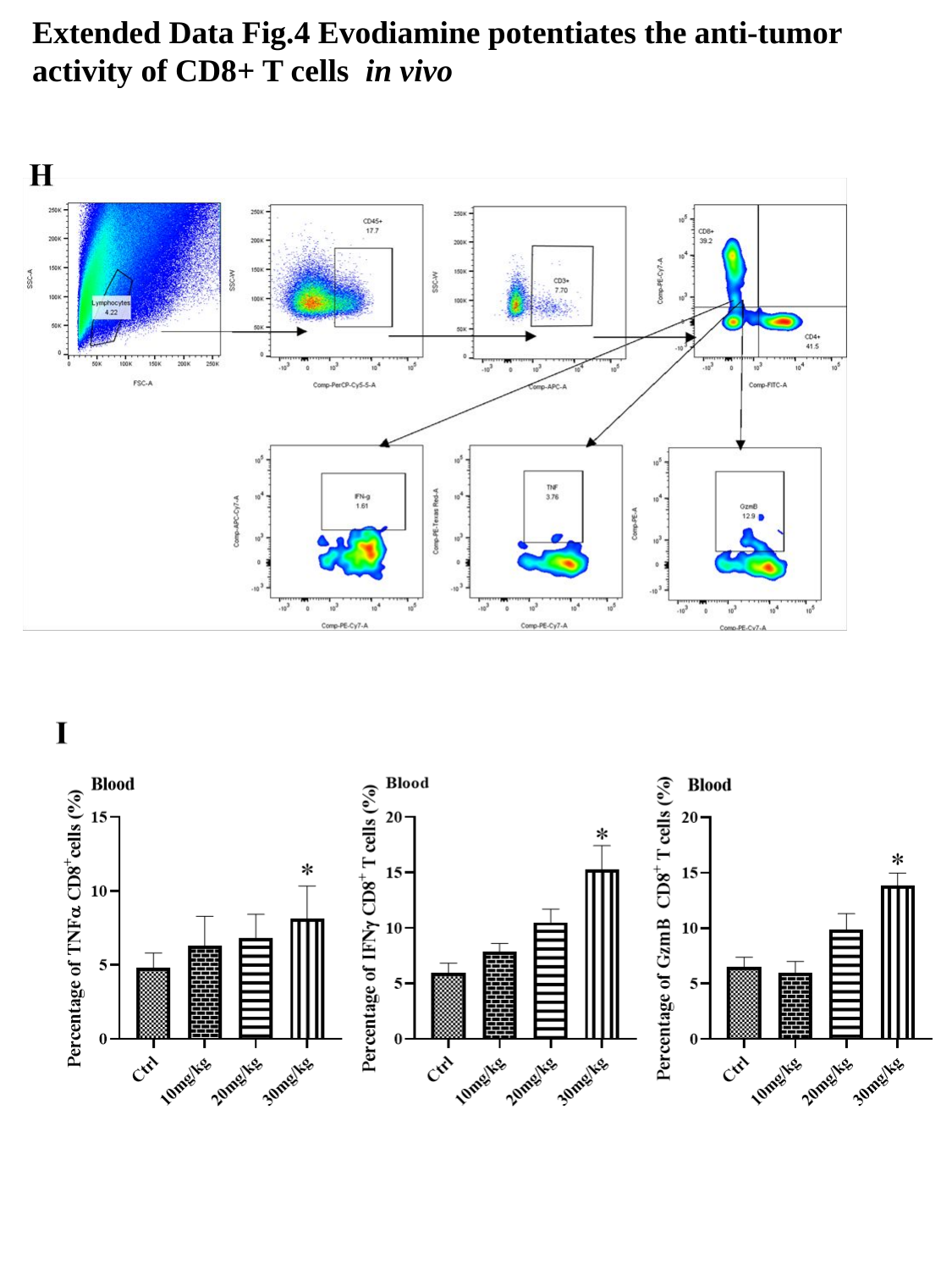

Extended Data Fig.4 Evodiamine potentiates the anti-tumor
activity of CD8+ T cells in vivo

## Slide 8
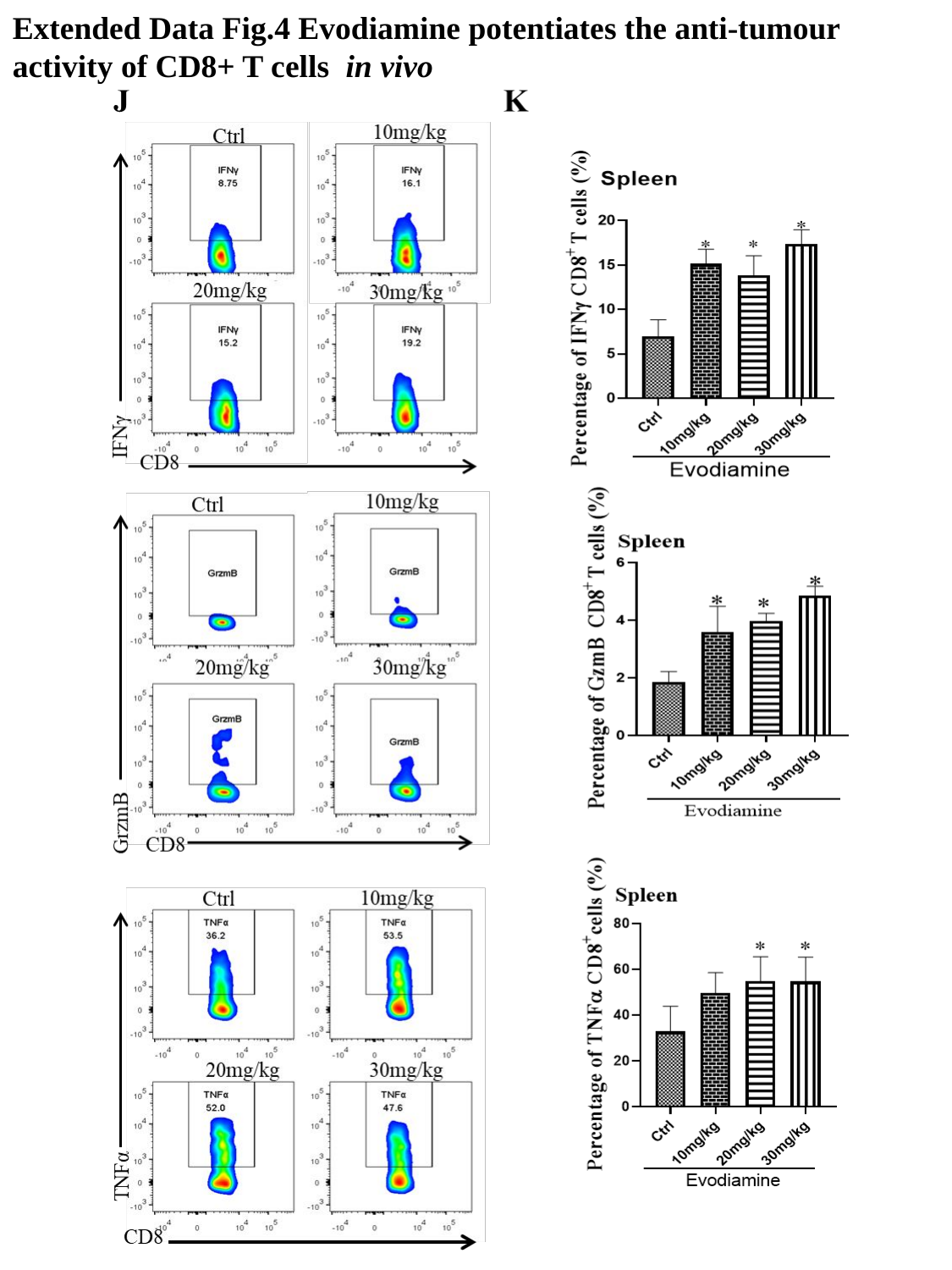

Extended Data Fig.4 Evodiamine potentiates the anti-tumour
activity of CD8+ T cells in vivo

## Slide 9
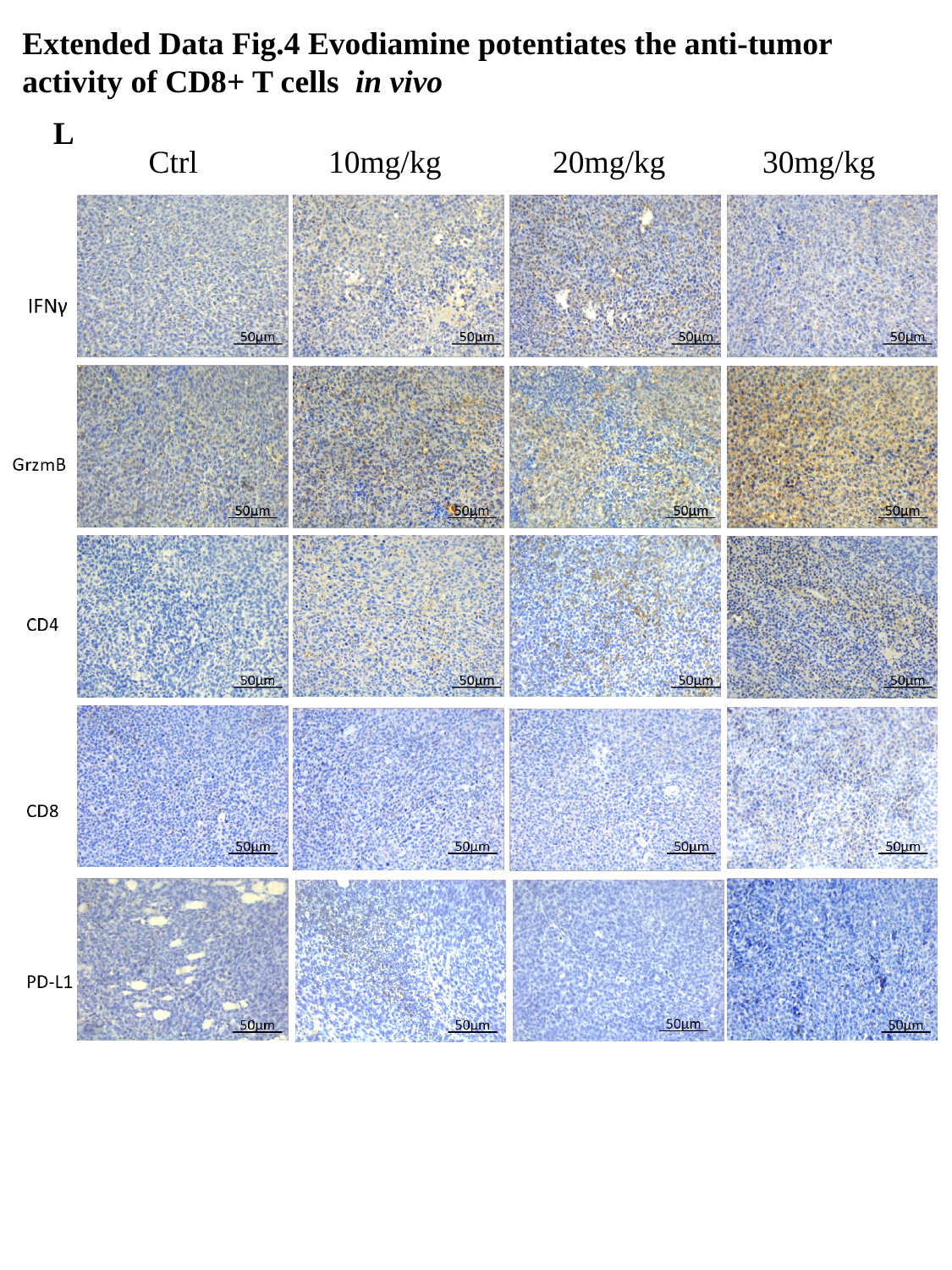

Extended Data Fig.4 Evodiamine potentiates the anti-tumor
activity of CD8+ T cells in vivo
L
Ctrl
10mg/kg
20mg/kg
30mg/kg

## Slide 10
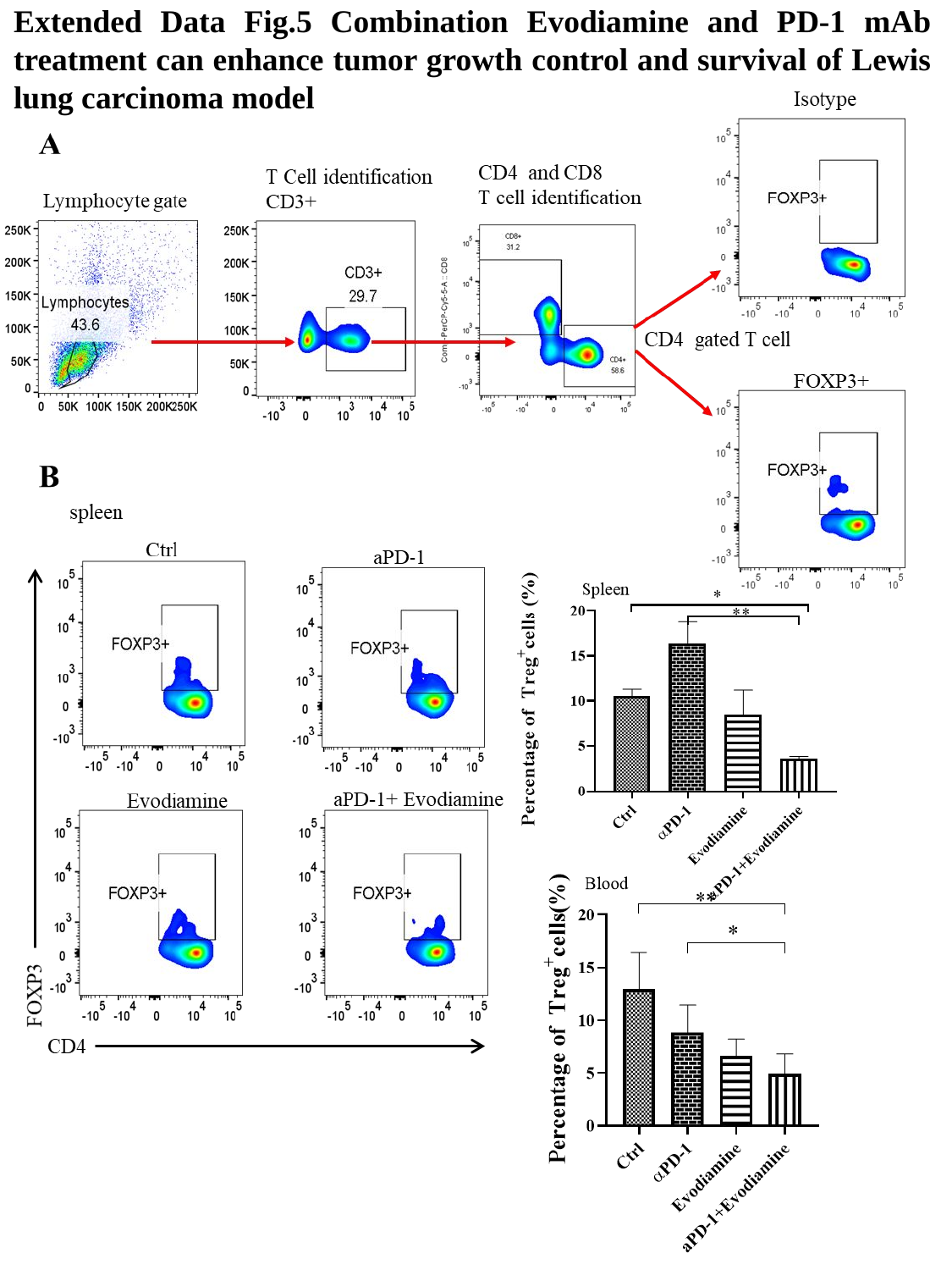

Extended Data Fig.5 Combination Evodiamine and PD-1 mAb treatment can enhance tumor growth control and survival of Lewis lung carcinoma model

## Slide 11
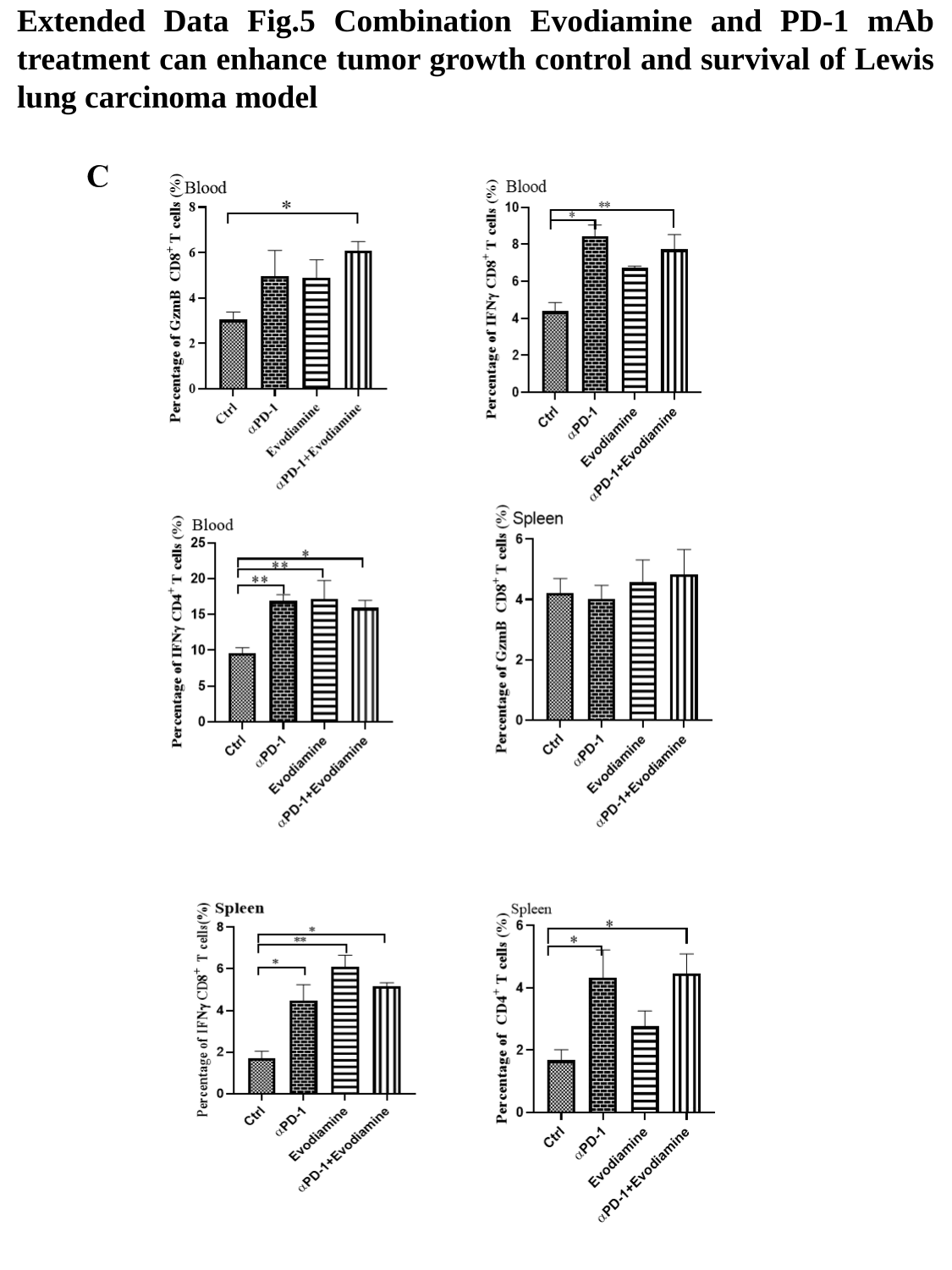

Extended Data Fig.5 Combination Evodiamine and PD-1 mAb treatment can enhance tumor growth control and survival of Lewis lung carcinoma model

## Slide 12
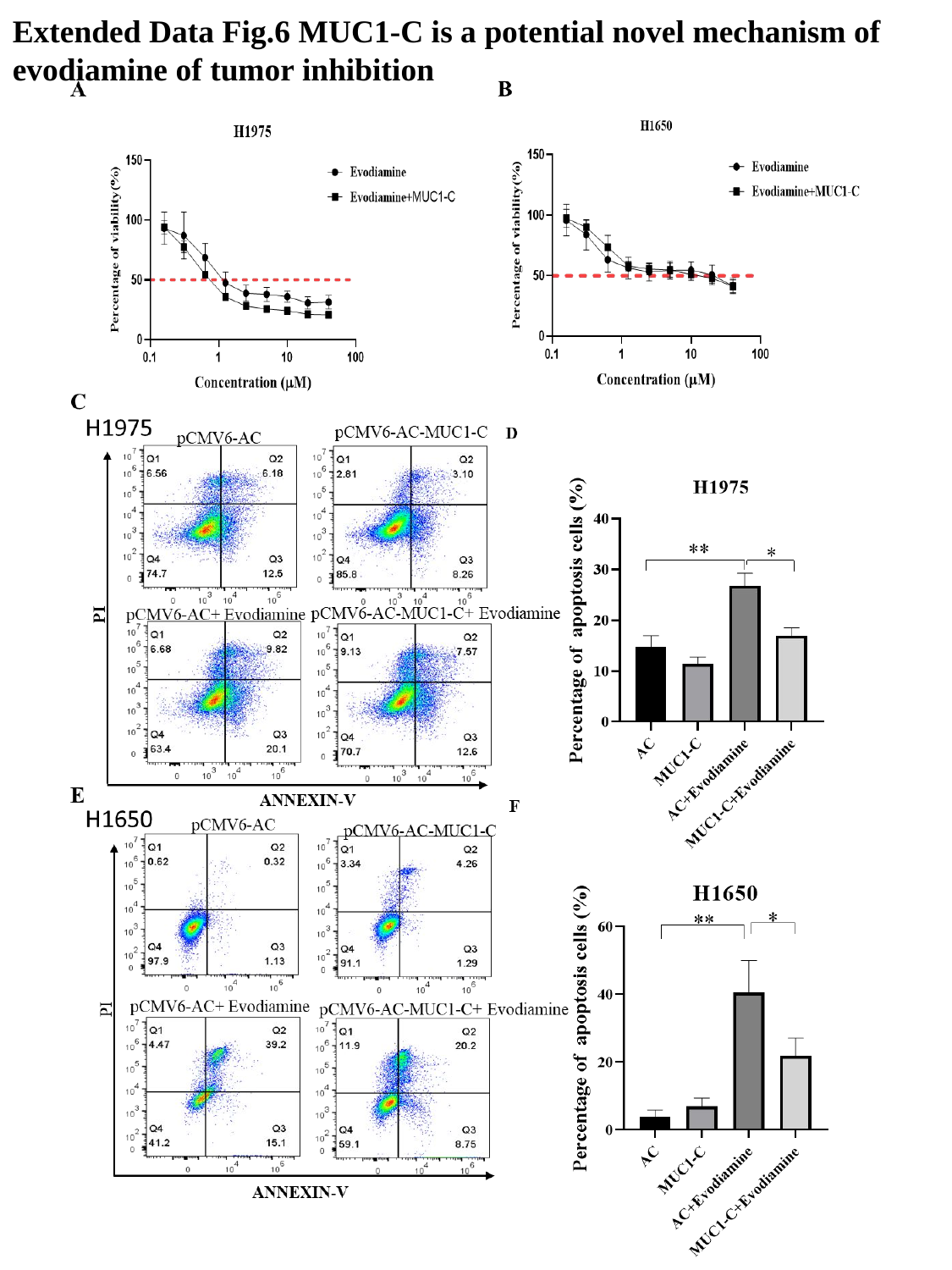

Extended Data Fig.6 MUC1-C is a potential novel mechanism of evodiamine of tumor inhibition
